# Supplementary material for: Translating Evidence for a Mediterranean-Style Dietary Pattern into Routine Care for Coronary Heart Disease and Type 2 Diabetes: Implementation and Evaluation in a Targeted Public Health Service in Australia
Source: Healthcare (Basel). 2025 Feb 26;13(5):506. doi: 10.3390/healthcare13050506 (PMC11898626; doi:10.3390/healthcare13050506)
Supplement: Supplementary file 1 [file healthcare-13-00506-s001.zip › healthcare-3478917-supplementary.pdf]

Additional file S1 – Supplementary tables

Translating Evidence for a Mediterranean-Style Dietary Pattern into Routine Care for Coronary Heart Disease and Type 2 Diabetes: Implementation and Evaluation in a Targeted Public Health Service in Australia

Hannah L. Mayr *, Lisa Hayes, William Y. S. Wang , Eryn Murray, Jaimon T. Kelly, Michelle Palmer and Ingrid J. Hickman

*Correspondence: hannah.mayr@health.qld.gov.au; Tel.: +61-7-3176-7938

Table S1. CFIR-ERIC Implementation Strategy Matching Tool top 15 prioritized ERIC implementation strategies and their definition

**Implementation strategy**[a]	**Definition**[b]
Identify and prepare champions	Identify and prepare individuals who dedicate themselves to supporting, marketing, and driving through an implementation, overcoming indifference or resistance that the intervention may provoke in an organization
Conduct educational meetings	Hold meetings targeted toward different stakeholder groups (e.g., providers, administrators, other organizational stakeholders, and community, patient/consumer, and family stakeholders) to teach them about the clinical innovation
Conduct local consensus discussions	Include local providers and other stakeholders in discussions that address whether the chosen problem is important and whether the clinical innovation to address it is appropriate
Inform local opinion leaders	Inform providers identified by colleagues as opinion leaders or "educationally influential" about the clinical innovation in the hopes that they will influence colleagues to adopt it
Assess for readiness and identify barriers and facilitators	Assess various aspects of an organization to determine its degree of readiness to implement, barriers that may impede implementation, and strengths that can be used in the implementation effort
Capture and share local knowledge	Capture local knowledge from implementation sites on how implementers and clinicians made something work in their setting and then share it with other sites
Create a learning collaborative	Facilitate the formation of groups of providers or provider organizations and foster a collaborative learning environment to improve implementation of the clinical innovation
Identify early adopters	Identify early adopters at the local site to learn from their experiences with the practice innovation
Involve patients/consumers and family members	Engage or include patients/consumers and families in the implementation effort
Conduct local needs assessment	Collect and analyse data related to the need for the innovation
Build a coalition	Recruit and cultivate relationships with partners in the implementation effort
Facilitation	A process of interactive problem solving and support that occurs in a context of a recognized need for improvement and a supportive interpersonal relationship
Develop educational materials	Develop and format manuals, toolkits, and other supporting materials in ways that make it easier for stakeholders to learn about the innovation and for clinicians to learn how to deliver the clinical innovation
Obtain and use patients/consumers and family feedback	Develop strategies to increase patient/consumer and family feedback on the implementation effort
Alter incentive/allowance structures	Work to incentivise the adoption and implementation of the clinical innovation

[a]Tool source: https://cfirguide.org/tools/

[b]Definitions sourced from: Powell BJ, et al. (2015) *Implement Sci* 10, 21.

Table S2. A summary of core Mediterranean-style or heart healthy dietary pattern principles; list provided in clinician survey prior to questions related to acceptability, feasibility and adoption

**Core Mediterranean-style or heart healthy dietary pattern principles**
Include plenty of vegetables and fruit every day
Choose wholegrain breads and cereals
Include nuts and seeds
Include fish or seafood
Include legumes or lentils
Include unflavoured milk, yoghurt and cheese
Have moderate intake of lean poultry and eggs
Limit intake of red meat
Use mostly extra virgin olive oil for dressing and cooking
Include other healthy fats such as avocado or other plant oils high in unsaturated fats
Focus on home cooking or food preparation
Make water the drink of choice
Only if you choose to drink alcohol, choose red wine (and only with meals)
Limit processed foods and sweets
Use herbs and spices to flavour foods instead of salt

Table S3. Clinician survey respondents self-reported diet-related roles and usual practices for patients with coronary heart disease and type 2 diabetes

Practice variable	Total cohort (n=57)	Dietitian (n=7)	Nurse/Diabetes Educator (n=29)	Doctor (n=15)	Other allied health (n=6)
Assessing or monitoring diet/any aspect of nutritional intake	41 (71.9)	7 (100.0)	26 (89.7)	7 (46.7)	1 (16.7)
Initiating any verbal advice/education related to diet	50 (87.7)	7 (100.0)	27 (93.1)	14 (93.3)	2 (33.3)
Responding to questions related to diet	50 (87.7)	7 (100.0)	27 (93.1)	14 (93.3)	2 (33.3)
Providing hardcopy education materials related to diet	37 (64.9)	7 (100.0)	22 (75.9)	8 (53.3)	0 (0.0)
Recommending/providing online education content (electronic factsheet, website or videos) related to diet	33 (57.9)	7 (100.0)	22 (75.9)	4 (26.7)	0 (0.0)
Recommending or demonstrating mobile apps for monitoring or intervention related to diet	25 (43.9)	6 (85.7)	14 (48.3)	5 (33.3)	0 (0.0)
Referral to and/or recommending patients see a dietitian	45 (78.9)	6 (85.7)*	23 (79.3)	12 (80.0)	4 (66.7)
Referral to and/or recommending patients access cardiac rehabilitation	28 (49.1)	4 (57.1)	16 (55.2)	6 (40.0)	2 (33.3)
Initiate discussions about diet with patients (n=50)					
Most of the time or always	32 (64.0)	Not asked	22 (75.8)	9 (60.0)	1 (16.7)
Sometimes	11 (22.0)		3 (10.3)	7 (40.0)	2 (33.3)
Rarely or never	7 (14.0)		4 (13.8)	0 (0.0)	3 (50.0)
Patients initiate discussions about diet (n=50)					
Most of the time or always	13 (26.0)	Not asked	11 (37.9)	2 (13.4)	0 (0.0)
Sometimes	26 (52.0)		14 (48.3)	8 (53.3)	4 (66.7)
Rarely or never	11 (22.0)		4 (13.7)	5 (33.3)	2 (33.3)

Data are n (%)

*The question specified if you are a dietitian this may include referring on outside your service

Table S4. Whether clinician's adoption of a Mediterranean-style or heart healthy dietary pattern approach in practice with relevant patients is associated with demographic or clinical characteristics

**Characteristics variable**	**Clinician response to question 'Advice would align to these dietary pattern principles'**			**P-value***
	**Most of the time or always**	**Sometimes**	**Rarely or never**	
Age				0.786
20 to 29 years	11 (91.7)	1 (8.3)	-	
30 to 39 years	11 (73.3)	3 (20.0)	1 (6.7)	
40 to 49 years	7 (77.8)	2 (22.2)	-	
50 to 59 years	11 (91.7)	1 (8.3)	-	
60 to 69 years	3 (100.0)	-	-	
Gender				<0.001*
Male	8 (57.1)	6 (42.9)	-	
Female	36 (94.7)	1 (2.6)	1 (2.6)	
Country of birth				0.211
Australia	33 (89.2)	3 (8.1)	1 (2.7)	
Outside Australia	12 (75.0)	4 (25.0)	-	
Duration in current health professional role				0.564
<1 to 10 years	23 (85.2)	3 (11.1)	1 (3.7)	
>10 years	24 (85.7)	4 (14.3)		
Duration working with coronary heart disease and/or type 2 diabetes patients				0.182
<1 to 10 years	19 (90.5)	1 (4.8)	1 (4.8)	
>10 years	28 (82.4)	6 (17.6)	-	

Data are n(%), reporting on 55 clinicians who completed the relevant survey question, except for age data n=51 and gender data n=52
*Statistical test is Chi-squared test, with significance at p<0.05

Table S5. Whether clinician survey respondents had utilised specific individual recommended education materials aligned to Mediterranean-style or heart healthy dietary pattern approach, including within target services and professions

Education materials	Total cohort (n=55)†	H1 Diabetes (n=13)	H1 Cardiology (n=23)	H2 Cardiology (n=13)	Community CDS (n=11)†	Dietitian (n=7)	Nurse/ Diabetes Educator (n=27)†	Doctor (n=15)	Other allied health (n=6)
At least one or more	45 (81.8)	13 (100.0)	18 (78.3)	10 (76.9)	8 (72.7)	7 (100.0)	23 (85.2)	12 (80.0)	3 (50.0)
Health service 2-page Mediterranean-style diet factsheet	29 (52.7)	11 (84.6)	8 (34.8)	10 (76.9)	6 (54.5)	7 (100.0)	13 (48.1)	9 (60.0)	0 (0.0)
NEMO 6-page Mediterranean-style diet factsheet	18 (32.7)	6 (46.2)	5 (21.7)	6 (46.2)	4 (36.4)	7 (100.0)	6 (22.2)	3 (20.0)	1 (16.7)
NEMO 4-page 'Eating for a healthy heart' factsheet	9 (16.4)	2 (15.4)	3 (13.0)	5 (38.5)	2 (18.2)	5 (71.4)	3 (11.1)	0 (0.0)	1 (16.7)
Heart Foundation heart healthy eating principles pictorial factsheet	18 (32.7)	3 (23.1)	8 (34.8)	4 (30.8)	5 (45.5)	2 (28.6)	10 (37.0)	4 (46.7)	2 (33.3)
Heart healthy eating content in My Heart My Life booklet*	18 (32.7)	0 (0.0)	12 (52.2)	3 (23.1)	4 (36.4)	1 (14.3)	12 (44.4)	3 (20.0)	2 (33.3)
Heart Foundation heart healthy eating sections on website	16 (29.1)	2 (15.4)	9 (39.1)	5 (30.5)	5 (45.5)	2 (28.6)	8 (29.6)	4 (46.7)	2 (33.3)
Heart Foundation heart healthy recipes (website or e-book)	16 (29.1)	4 (30.8)	8 (34.8)	4 (30.8)	5 (45.5)	5 (71.4)	9 (33.3)	1 (6.7)	1 (16.7)
Oldways website on Mediterranean-style diet	3 (5.5)	1 (7.7)	1 (4.3)	1 (7.7)	1 (9.1)	2 (28.6)	1 (3.7)	0 (0.0)	0 (0.0)
Baker HDI factsheet on healthy convenience meals	18 (32.7)	10 (76.9)	4 (17.4)	4 (30.8)	3 (27.3)	7 (100.0)	8 (29.6)	3 (20.0)	0 (0.0)
Vimeo webpage with heart healthy eating video/s**	1 (1.8)	0 (0.0)	0 (0.0)	1 (7.7)	0 (0.0)	1 (14.3)	0 (0.0)	0 (0.0)	0 (0.0)

Data are n (%)

†data missing for 2 participants (1 nurse, 1 diabetes educator) with incomplete surveys

*Booklet published by Heart Foundation typically provided to patients with acute coronary syndrome

**In response to clinician feedback webpage of videos was only created and shared October 2022 and relied on clinical champions to share

H1, Hospital 1; H2, Hospital 2; CDS, Chronic Disease Service; NEMO, Nutrition Education Materials Online (state government dietitian created resources); Baker HDI, Baker Heart and Diabetes Institute
